# Comparison between two thoracotomy closure techniques: postoperative pain and pulmonary function*

**DOI:** 10.1590/S1806-37132014000400006

**Published:** 2014

**Authors:** Juliana Duarte Leandro, Olavo Ribeiro Rodrigues, Annie France Frere Slaets, Aurelino F. Schmidt, Milton L. Yaekashi

**Affiliations:** University of Mogi das Cruzes, Mogi das Cruzes, Brazil; Department of Thoracic Surgery, University of Mogi das Cruzes Mogi das Cruzes, Brazil; Graduate Program, University of Mogi das Cruzes, Mogi das Cruzes, Brazil; Department of Thoracic Surgery, Hospital das Clínicas Luzia de Pinho Melo, Mogi das Cruzes (SP) Brasil; Department of Thoracic Surgery, Hospital das Clínicas Luzia de Pinho Melo, Mogi das Cruzes (SP) Brasil

**Keywords:** Thoracic surgery, Suture techniques, Acute pain

## Abstract

**OBJECTIVE:**

To compare two thoracotomy closure techniques (pericostal and transcostal suture) in terms of postoperative pain and pulmonary function.

**METHODS:**

This was a prospective, randomized, double-blind study carried out in the Department of Thoracic Surgery of the Luzia de Pinho Melo *Hospital das Clínicas *and at the University of Mogi das Cruzes, both located in the city of Mogi das Cruzes, Brazil. We included 30 patients (18-75 years of age) undergoing posterolateral or anterolateral thoracotomy. The patients were randomized into two groups by the type of thoracotomy closure: pericostal suture (PS; n = 16) and transcostal suture (TS; n = 14). Pain intensity during the immediate and late postoperative periods was assessed by a visual analogic scale and the McGill Pain Questionnaire. Spirometry variables (FEV_1_, FVC, FEV_1_/FVC ratio, and PEF) were determined in the preoperative period and on postoperative days 21 and 60.

**RESULTS:**

Pain intensity was significantly greater in the PS group than in the TS group. Between the preoperative and postoperative periods, there were decreases in the spirometry variables studied. Those decreases were significant in the PS group but not in the TS group.

**CONCLUSIONS:**

The patients in the TS group experienced less immediate and late post-thoracotomy pain than did those in the PS group, as well as showing smaller reductions in the spirometry parameters. Therefore, transcostal suture is recommended over pericostal suture as the thoracotomy closure technique of choice.

## Introduction

Conventional thoracic surgery can cause several complications, because access to the pleural cavity requires sectioning of the intercostal muscles, opening of the parietal pleura, and spreading of the ribs. In this procedure, the costal periosteum and the intercostal neurovascular bundle can suffer injuries of varying degrees, resulting from the mechanical effects of retractors or the thermal effects of electrocautery.^(^ 1 ^-^ 4 ^)^

Most patients undergoing thoracotomy complain of pain, which is responsible for shallow breathing, with a consequent decrease in lung volumes and capacities, as well as secretion retention and atelectasis.^(^ 5 ^-^ 8 ^)^ To prevent the acute pain and respiratory changes that accompany thoracic interventions, new approaches have been used, such as minimally invasive thoracotomy. The advent of video-assisted surgery two decades ago enabled the use of smaller access ports to the thoracic cavity and resection via small thoracotomy. This reduced the incidence of postoperative pain and the changes in pulmonary function.^(^ 2 ^,^ 9 ^)^ However, conventional techniques for thoracic surgery cannot always be replaced by minimally invasive techniques, and, in such cases, acute and/or chronic pain may be present. There are still many resection cases requiring major posterolateral or anterolateral thoracotomy, especially in patients with tumors and in those with chronic infectious diseases. These major surgical procedures require some precautions, especially during thoracotomy closure, because, in practice, intercostal space closure is commonly performed with sutures around the ribs, designated pericostal sutures (PSs).

Thoracotomy closure with PSs may cause injury due to compression of the neurovascular bundle, which courses on the lower edge of the rib, as a result of its anatomical position. The structure most vulnerable to trauma is the cutaneous branch of the intercostal nerve, because of its location on the costal margin. Its trauma due to compression or crushing during the procedure of costal approximation implies pain and cutaneous paresthesia for some days or months postoperatively.^(^ 8 ^)^

In an attempt to minimize pain, some thoracic surgeons are currently replacing PS with transcostal suture (TS), which consists in passing the approximation suture through holes drilled directly into the ribs. This technique has shown positive and promising results regarding decreased pain in the postoperative period.^(^ 10 ^-^ 12 ^)^

The objective of the present study was to compare two thoracotomy closure techniques, i.e., PS and TS, in terms of postoperative pain and pulmonary function.

## Methods

This was a prospective, randomized, double-blind study carried out between August of 2011 and September of 2012. The study project was approved by the Research Ethics Committee of the University of Mogi das Cruzes on November 18, 2010 (Protocol no. 150/2010, CAAE 0144.0.0237.000-10).

We included all patients (18-75 years of age) undergoing posterolateral or anterolateral thoracotomy through intracavitary access. The exclusion criteria were as follows: having bone metastasis; having a history of pain caused by other comorbidities; and being dependent on drugs, opioid analgesics, or any other substance that affects one's sensitivity to pain. 

The patients were randomized into two groups by the type of thoracotomy closure: PS group and TS group. To that end, we used web-based randomization. 

**Figure 1:**
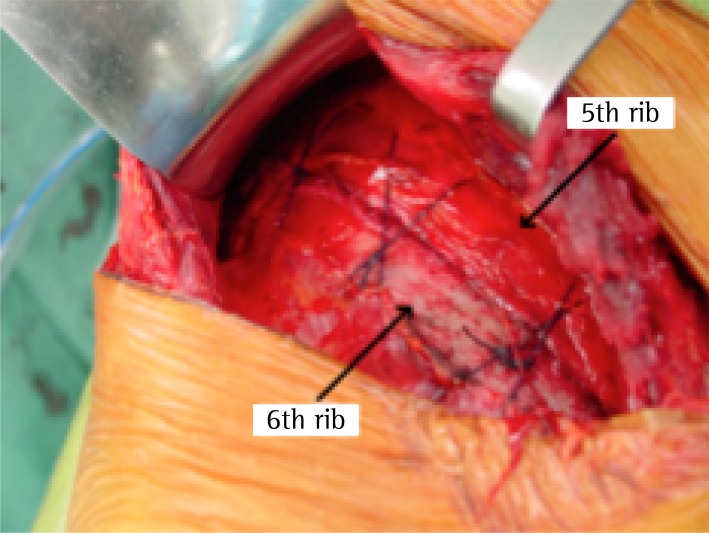
Closure technique with pericostal suture.

In the PS group, thoracotomy closure was performed by passing the suture around the fifth rib, close to its upper border, and around the sixth rib, away from its lower border, and drawing them together (Figure 1).

**Figure 2:**
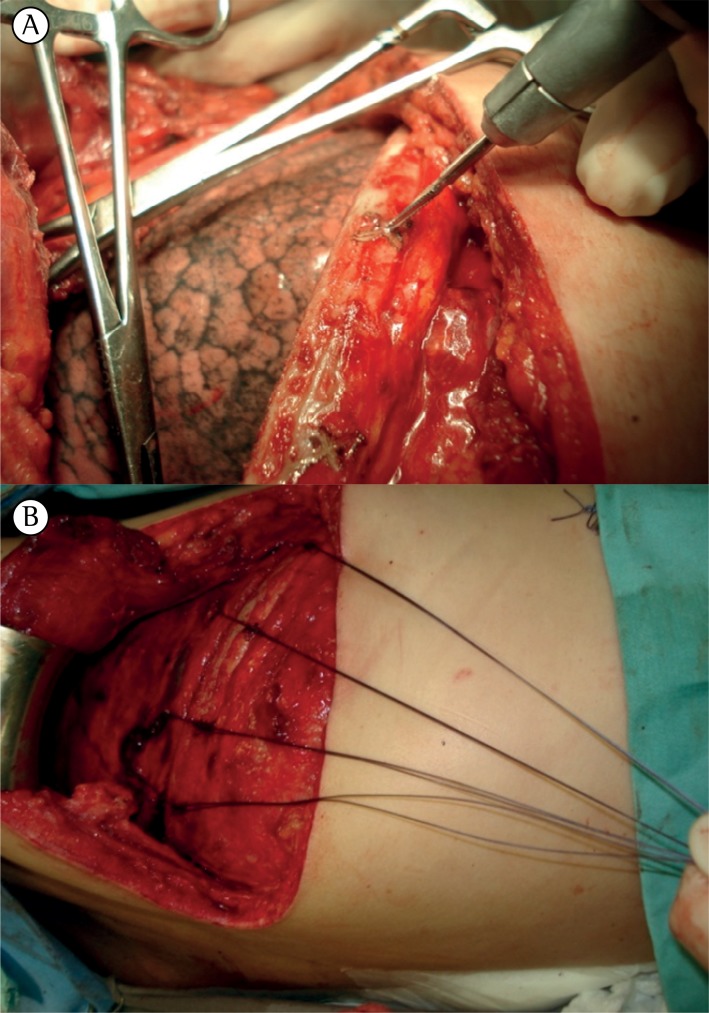
In A, rib drilling. In B, approximation of the fifth and sixth ribs after the suture was passed through the drill holes.

In the patients in the TS group, closure was performed as follows. The position for the suture drill holes was marked on the periosteum using an electrocautery knife. Subsequently, holes were drilled into the fifth and sixth ribs using a 7-mm diameter drill, which was rotated by a dental motor (LB100; Beltec, Araraquara, Brasil; Figures 2A and 2B). Four equidistant holes were drilled into each rib. The sutures were passed through the drill holes, and transcostal closure was performed (Figure 2B).

### All closures were performed using coated synthetic absorbable polyglactin 910 suture (VICRYL**(r)**, Ethicon Endo-Surgery, Inc. Cincinnati, OH, USA), size 1, and a circular needle (40 mm).

The study variables were postoperative pain and pulmonary function as assessed by spirometry on postoperative day (POD) 21 and POD 60 for comparisons with the values obtained in the preoperative period. According to the study protocol, pain was assessed from POD 1 to POD 10, as well as in the late postoperative period (POD 21 and POD 60). 

To assess pain, we used a one-dimensional visual analog scale (VAS) and the McGill Pain Questionnaire.^(^ 13 ^)^ The VAS is a 0 to 10 point scale, with 0 meaning complete absence of pain and 10 meaning the greatest level of experienced pain, with which therapists ask patients about their pain intensity. The McGill Pain Questionnaire assesses pain in four distinct domains (sensory, affective, evaluative, and mixed), on the basis of words, designated descriptors, which patients select to describe their pain.^(^ 13 ^)^ Patients are instructed to choose, from among 20 groups of descriptors, those that best describe their pain at the time of the assessment.^(^ 13 ^)^ The first 10 descriptors are related to the sensory dimension of pain. Descriptors 11 to 15 are related to the affective dimension of pain. Descriptor 16 addresses pain in an evaluative way, whereas descriptors 17 to 20 represent a mixed class of alternative words.^(^ 13 ^)^

Spirometry was performed in accordance with the American Thoracic Society 1995 criteria and the Brazilian Thoracic Association criteria.^(^ 14 ^)^ In a stable setting, the patient sat in a comfortable position and, wearing a nose clip, performed a maximal forced expiratory maneuver, from TLC to RV. Thus, FVC, FEV_1_, FEV_1_/FVC ratio, and PEF were measured.^(^ 14 ^)^

Individual data are expressed as mean and standard error. Statistical analysis was performed using GraphPad Instant Software (GraphPad Software, San Diego, CA, USA). Categorical variables (gender, race, clinical diagnosis, and surgical procedure) were assessed by the chi-square test. For numerical variables (spirometry), we used the Student's t-test to compare results between the PS and TS groups and one-way ANOVA to compare preoperative and postoperative results within the same group. For the analysis of pain as measured by the VAS, we used the Student's t-test, whereas, for the analysis of pain as determined by the McGill Pain Questionnaire, we used the Mann-Whitney test.^(^ 15 ^)^ The level of significance set for rejection of the null hypothesis was p < 0.05.^(^ 15 ^)^

## Results

We included 31 patients, of whom 16 and 15 were randomized to the PS and TS groups, respectively. Only 1 patient in the TS group did not return for reassessment and was excluded from the study. Table 1 shows the characteristics of the sample.

**Table 1:** Sample characteristics and procedures performed in the groups studied.a

Variables	Groups		p
	Pericostal suture	Transcostal suture	
	(n = 16)	(n = 14)	
Age, years^b^	53.6 ± 3.4	48.9 ± 4.4	0.39
Gender			
Male	11 (68.8)	7 (50.0)	0.50
Female	5 (31.3)	7 (50.0)	
Race			
White	14 (87.5)	10 (71.4)	0.14
Afro-descendent	2 (12.5)	3 (21.4)	
Asian	0 (0.0)	1 (7.1)	
Surgery performed			
Lobectomy	9 (56.35)	8 (57.1)	0.12
Bilobectomy	4 (25.0)	3 (21.4)	
Segmentectomy	3 (18.8)	3 (21.4)	

a Values expressed as n (%), except where otherwise indicated

b Values expressed as mean ± SE.

The diagnosis of the patients in the PS and TS group was, respectively, as follows: adenocarcinoma, in 10 and 8 patients; epidermoid carcinoma, in 3 and 3; small cell carcinoma, in 2 and 3; and tuberculosis sequelae, in 1 and 0. Lobectomy was the most commonly performed surgical procedure (Table 1). The mean surgical time was 271.5 ± 25.7 min for the PS group and 250.3 ± 23.4 min for the TS group (p = 0.88).

**Figure 3:**
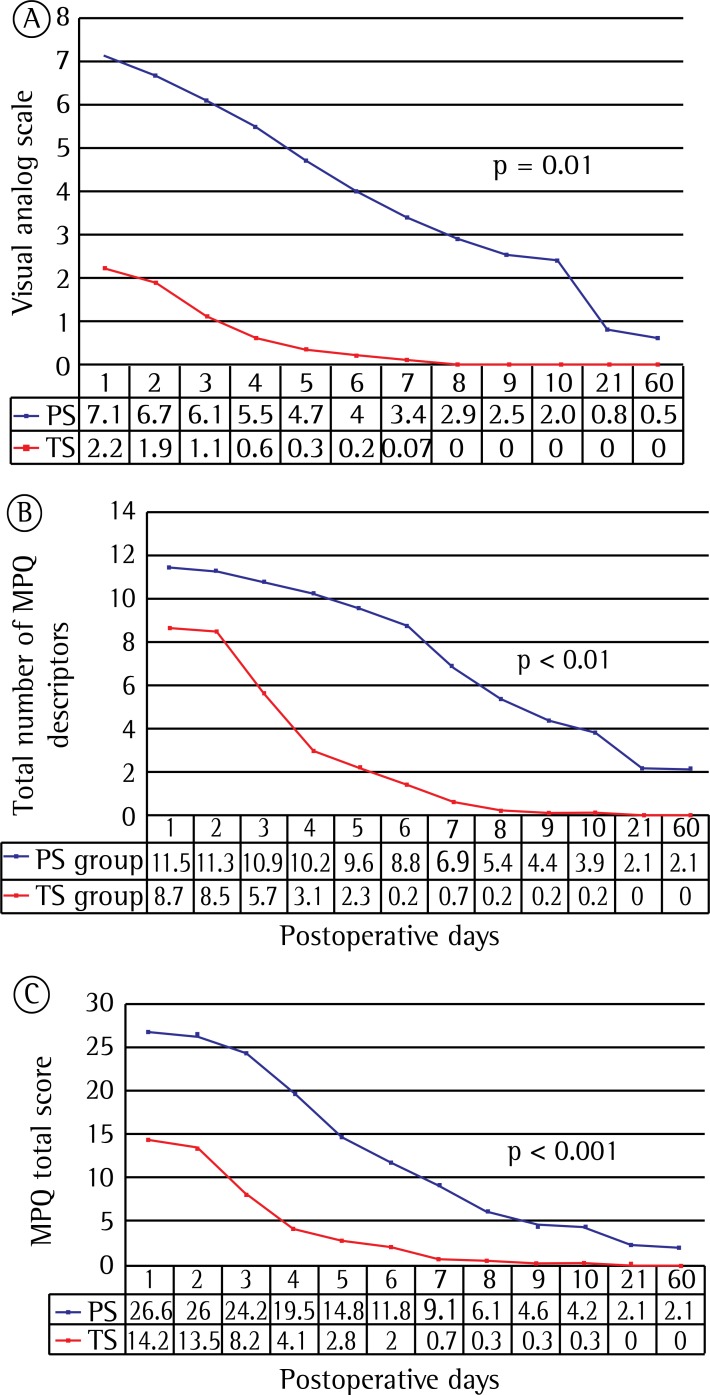
Comparison of mean pain intensity on various postoperative days in the pericostal suture (PS) and transcostal suture (TS) groups. In A, visual analog scale. In B, total number of McGill Pain Questionnaire (MPQ) descriptors selected by the patients. In C, MPQ total score.

Mean pain intensity (values expressed as n) was calculated for each POD. In both groups, there was a reduction in pain intensity in the follow-up period. Pain intensity was greater for the patients in the PS group than for those in the TS group from the immediate postoperative period, and this difference was statistically significant until POD 7 (p < 0.0001; Figure 3A). For the patients in the TS group, pain was minimal or absent around POD 7, whereas the patients in the PS group still reported moderate pain at that time point. In the PS group, pain was reported as minimal only on POD 60. After the McGill Pain Questionnaire was administered, we calculated and compared the mean total numbers of descriptors chosen and the mean questionnaire total scores for each assessment day. This assessment was performed from POD 1 to POD 10 and repeated on POD 21 and POD 60. 

When we compared the total numbers of descriptors chosen by the patients, we found that they were greater in the PS group than in the TS group. Postoperative pain intensity as assessed by this scale was found to be greater for the patients in the PS group. This difference was statistically significant between the two groups until POD 10 (p < 0.01; Figure 3B). When we compared the questionnaire total scores, we found that they were higher in the PS group than in the TS group, with this difference being statistically significant for the first 10 PODs (p < 0.001; Figure 3C).

Spirometric assessment of pulmonary function was performed at three distinct time points: in the preoperative period; on POD 21; and on POD 60. The surgical procedure led to a reduction in spirometric values in both groups, because the surgical procedure results in partial resection of the lung; however, according to the statistical analysis, the sample was homogeneous in terms of the type of surgery performed (p = 0.12). Table 2 shows the spirometric values at each time point of the study in the two groups. The FVC, FEV_1_ and PEF values were significantly lower postoperatively than preoperatively in the PS group, whereas there were no significant differences in these values in the TS group.

**Table 2:** Spirometry results in the preoperative period and on postoperative days 20 and 60 in the groups studied.a

Variable	Time point	PS group	p	TS group	p
FVC, L	Pre	3.00 ± 0.30	0.007	2.85 ± 0.20	0.14
	POD 21	2.10 ± 0.10		2.38 ± 0.20	
	POD 60	2.26 ± 0.10		2.61 ± 0.30	
					
FEV_1_, L	Pre	2.48 ± 0.10	0.01	2.33 ± 0.30	0.28
	POD 21	1.72 ± 0.10		1.91 ± 0.30	
	POD 60	1.89 ± 0.10		2.13 ± 0.30	
					
PEF, L/s	Pre	5.96 ± 0.50	0.02	5.30 ± 0.60	0.29
	POD 21	4.03 ± 0.40		4.41 ± 0.50	
	POD 60	4.80 ± 0.50		5.19 ± 0.70	
					
FEV_1_/FVC, %	Pre	83.4 ± 2.0	0.71	79.8 ± 4.0	0.51
	POD 21	82.5 ± 2.0		83.2 ± 3.0	
	POD 60	84.1 ± 2.0		81.7 ± 3.0	

PS : pericostal suture

TS : transcostal suture

Pre : preoperative period

POD : postoperative day

a Values expressed as mean ± SE.

## Discussion

Confirming the interest in the subject, during the present study, a systematic review was published on thoracotomy closure techniques and their relationship with post-thoracotomy pain.^(^ 16 ^)^ The authors of that review, searching the Cochrane Plus Library using the search terms "pain", "thoracotomy", and "suture", found 174 publications that linked the surgical technique employed with postoperative pain. Of those, 11 publications met the selection criteria established for that review, and, of those 11, 6 compared the thoracotomy closure technique with post-thoracotomy pain, only 4 of which were randomized studies. Those authors concluded that there is a need for further scientific evidence on certain technical aspects of thoracotomy closure techniques and their relationship with post-thoracotomy pain. In addition, they pointed out that only through the development of prospective randomized studies specifically comparing the different thoracotomy closure techniques described in the literature and assessing their relationship with post-thoracotomy pain will it be possible to make recommendations in this regard. However, the review made clear that a necessary aspect for reducing post-thoracotomy pain, and that should be common to all closure techniques, is a focus on intercostal nerve preservation.

Among the variables selected for the present study, pain was the one that caused the most difficulty in terms of assessment, because it is a continuous variable that is difficult to quantify. In addition to being a symptom, it is a subjective experience and is influenced by various factors, such as environmental, emotional, behavioral, and social factors. Therefore, we used two standard tools (a VAS and the McGill Pain Questionnaire).^(^ 13 ^)^

The largest prospective study of this subject included 280 patients undergoing posterolateral thoracotomy, divided into two groups: TS (n = 140) and PS (n = 140).^(^ 10 ^)^ Pain was assessed by a numeric pain scale and the McGill Pain Questionnaire. Those instruments were administered in the second postoperative week, as well as in the first, second, and third postoperative months. The authors concluded that the patients treated with TS experienced less pain than did those undergoing PS. Although that study was not randomized, it had a consistent level of evidence to recommend the use of TS in thoracotomy closure.^(^ 10 ^)^

In an experimental study in dogs, pain was assessed in the immediate postoperative period following thoracotomy in 13 animals.^(^ 17 ^)^ Seven animals underwent closure with PS close to the lower border of the lower rib, compressing the (caudal) neurovascular bundle, and 6 dogs underwent closure with TS. Pain was assessed using pain threshold scores, which were based on parameters such as HR and RR, for a period of 24 h. The study showed that the animals treated with TS experienced significantly less pain.^(^ 17 ^)^ Although that experimental study used a similar methodology in terms of the surgical technique employed, which proved of great value in preventing compression of and injury to the intercostal nerve, its limitation was that it assessed pain only in the immediate postoperative period.^(^ 17 ^)^

The present study showed, through the use of the VAS and the McGill Pain Questionnaire, that the patients in the TS group experienced less pain than did those in the PS group; these results are similar to those reported in previous studies.^(^ 10 ^,^ 17 ^)^

In previous studies,^(^ 12 ^,^ 18 ^,^ 19 ^)^ thoracotomy closure was also performed using TS; however, there was variation in the technique used to open the intercostal space during access to the pleural cavity, which means that their results are not comparable to the results of the present study or to those of another study,^(^ 10 ^)^ in which technical variation in performing the thoracotomy involved harvesting of intercostal muscle flaps to protect the neurovascular bundle from the chest retractor. Therefore, the assessment of pain threshold in the postoperative period was impaired when comparing the transcostal and pericostal closure groups because there were different interventions.

The use of Finochietto retractors during chest opening is known to be responsible for much of the pain after the surgical procedure. In our study, we took this into account, which is why the same method for opening the chest wall was used in both groups, i.e., there was no variation in the technique for opening the chest wall, as previously suggested by other authors.^(^ 12 ^,^ 16 ^)^

The present study found that the patients in the PS group used a large number of descriptors to characterize their postoperative pain-on average, 11 descriptors on POD 1, with a mean score of 26. This has also been observed in a prospective study^(^ 6 ^)^ comparing pain, as assessed by the McGill Pain Questionnaire, in 40 patients undergoing either posterolateral thoracotomy or sternotomy. The mean number of descriptors used by the patients in the group undergoing posterolateral thoracotomy was 16, with a mean score of 30, values that are very close to those found in the present study.

Regarding pulmonary function, we observed that the patients undergoing standard thoracotomy closure (PS group) showed significantly lower FVC, FEV_1_, and PEF on POD 21 than in the preoperative period. These results are historically expected in the postoperative period after thoracotomy and were similar to those reported in previous studies.^(^ 20 ^,^ 21 ^)^

A previous study^(^ 19 ^)^ investigated pulmonary function in 16 patients after major thoracotomy. Spirometry was performed on POD 14. The authors observed that FVC, FEV_1_, and PEF were significantly lower postoperatively than preoperatively.^(^ 19 ^)^ Patient recovery in terms of these variables was due to improvement in ventilatory capacity, reduction of the chest wall injury caused by the surgical procedure, and pain relief.

A prospective study of 33 patients undergoing thoracic surgery evaluated the impact of lung resection on pulmonary function in lung cancer patients undergoing thoracotomy.^(^ 21 ^)^ Spirometry was performed in the preoperative period and in the sixth postoperative month. The FEV_1_, PEF, and FVC values statistically significantly decreased relative to the values obtained in the preoperative period. Such results were expected and are related to the direct impact of surgical resection and to postoperative pain.^(^ 21 ^)^

In the present study, we expected a decrease in the spirometry variables, because the surgical procedures involve resection of lung parenchyma. However, the procedures performed in both groups were quite similar, and less postoperative pain in the TS group translated into a smaller decrease in FVC, FEV_1_, and PEF. In the PS group, in which pain was found to be more severe, the decreases in the spirometry values were greater.

In conclusion, the patients undergoing closure of a posterolateral or anterolateral thoracotomy with TS experienced a significant decrease in immediate and late postoperative pain when compared with those undergoing closure with PS. In addition, the patients in the TS group showed smaller reductions in the spirometry parameters. Therefore, TS is recommended over PS as the thoracotomy closure technique of choice.
