# Supplementary material for: Comparison between two thoracotomy closure techniques: postoperative pain and pulmonary function
Source: J Bras Pneumol. 2014 Jul-Aug;40(4):389–96. doi: 10.1590/S1806-37132014000400006 (PMC4201169; doi:10.1590/S1806-37132014000400006)
Supplement: Translation

# Translation: Comparação entre duas técnicas de fechamento de toracotomia: dor pós-operatória e função pulmonar*

**Authors:** Juliana Duarte Leandro, Olavo Ribeiro Rodrigues, Annie France Frere Slaets, Aurelino F. Schmidt, Milton L. Yaekashi

**Affiliations:** Universidade de Mogi das Cruzes, Mogi das Cruzes (SP) Brasil; Universidade de Mogi das Cruzes, Mogi das Cruzes (SP) Brasil; Programa de Pós-Graduação, Universidade de Mogi das Cruzes, Mogi das Cruzes (SP) Brasil; Serviço de Cirurgia Torácica, Hospital das Clínicas Luzia de Pinho Melo, Mogi das Cruzes (SP) Brasil; Serviço de Cirurgia Torácica, Hospital das Clínicas Luzia de Pinho Melo, Mogi das Cruzes (SP) Brasil

**Keywords:** Cirurgia torácica, Técnicas de sutura, Dor aguda

## Abstract

**OBJETIVO:**

Comparar duas técnicas de fechamento de toracotomias (sutura pericostal e transcostal) em relação à dor pós-operatória e função pulmonar.

**MÉTODOS:**

Estudo prospectivo, randomizado e duplo-cego realizado no Serviço de Cirurgia Torácica do Hospital das Clínicas Luzia de Pinho Melo e na Universidade de Mogi das Cruzes, na cidade de Mogi das Cruzes, Brasil. Foram incluídos no estudo 30 pacientes submetidos a toracotomias posterolaterais ou anterolaterais, com idade entre 18 e 75 anos. Os pacientes foram randomizados em dois grupos em função do tipo de fechamento da toracotomia: sutura pericostal (SP; n = 16) e sutura transcostal (ST; n = 14). A intensidade da dor no pós-operatório imediato e tardio foi avaliada por uma escala visual analógica e questionário de dor McGill. Foram avaliadas variáveis espirométricas (VEF_1_, CVF, relação VEF_1_/CVF e PFE) no pré-operatório e nos 21º e 60º dias pós-operatórios.

**RESULTADOS:**

A intensidade da dor foi significativamente maior no grupo SP que no grupo ST. No grupo SP, houve reduções significativas nas variáveis espirométricas estudadas entre o período pré-operatório e pós-operatório. Essas reduções não foram significativas no grupo ST.

**CONCLUSÕES:**

Os pacientes no grupo ST apresentaram menor intensidade de dor pós-toracotomia, tanto imediata como tardia, e menor redução nos parâmetros espirométricos que os no grupo SP. Dessa forma, a técnica de fechamento de toracotomia por sutura transcostal é recomendada por apresentar vantagens sobre a técnica pericostal tradicional.

## Introdução

A cirurgia torácica convencional é passível de várias complicações, pois o acesso à cavidade pleural demanda a secção dos músculos intercostais, a abertura da pleura parietal e o afastamento das costelas. Nesse procedimento, o periósteo costal e o feixe neurovascular intercostal podem sofrer lesões de graus variáveis decorrentes da ação mecânica de afastadores ou pela ação térmica de eletrocautério.^(^ 1 ^-^ 4 ^)^

A maioria dos pacientes submetidos a toracotomia queixa-se de dor, sendo essa responsável pela respiração superficial, com consequente diminuição dos volumes e capacidades pulmonares, assim como retenção de secreção e atelectasia. ^(^ 5 ^-^ 8 ^)^ Com o objetivo de prevenir a dor aguda e as alterações respiratórias ocasionadas pelas intervenções torácicas, novas abordagens vêm sendo praticadas, como a toracotomia minimamente invasiva. O advento da cirurgia videoassistida nas duas últimas décadas possibilitou a prática de acessos menores à cavidade torácica com ressecções por toracotomias econômicas. Isso reduziu a incidência da dor pós-operatória e as alterações da função pulmonar.^(2,9) ^Entretanto, nem sempre as técnicas cirúrgicas convencionais torácicas podem ser substituídas pelas técnicas minimamente invasivas, e, nesses casos, a dor aguda e/ou crônica podem se tornar presentes. Existem ainda muitas ressecções que exigem a prática de toracotomias posterolaterais e anterolaterais amplas, principalmente em tumores e em doenças infecciosas crônicas. Esses procedimentos cirúrgicos amplos exigem alguns cuidados, principalmente durante o fechamento da toracotomia, pois é comum, na prática, realizar-se a aproximação dos espaços intercostais por meio de suturas que circundam as costelas, denominadas de suturas pericostais.

Ao realizar-se esse tipo de fechamento torácico, é possível que ocorra lesão por compressão do feixe neurovascular, presente na borda inferior da costela, em decorrência de sua posição anatômica. A estrutura mais vulnerável ao trauma é o ramo cutâneo do nervo intercostal, devido a sua localização na borda costal. Seu trauma por compressão ou esmagamento durante o procedimento de aproximação costal implicará no surgimento de dor e parestesia cutânea no período pós-operatório por alguns dias ou meses.^(^ 8 ^)^

Na tentativa de minimizar a dor, alguns cirurgiões torácicos atualmente vêm substituindo as suturas pericostais por suturas transcostais, que consistem na passagem dos fios de aproximação por furos realizados diretamente nos arcos costais. Essa técnica tem demonstrado resultados positivos e promissores quanto a diminuição da dor no período pós-operatório.^(^ 10 ^-^ 12 ^)^

O objetivo do presente estudo foi comparar duas técnicas de fechamento de toracotomias, através de sutura pericostal (SP) ou sutura transcostal (ST), em relação à dor pós-operatória e função pulmonar.

## Métodos

Trata-se de um estudo prospectivo, randomizado e duplo-cego realizado entre o período de agosto de 2011 e setembro de 2012. O projeto do estudo teve a aprovação do Comitê de Ética em Pesquisa da Universidade de Mogi das Cruzes em 18 de novembro de 2010, sob o número de processo 150/2010, CAAE 0144.0.0237.000-10.

Foram incluídos no estudo todos os pacientes submetidos a toracotomias posterolaterais ou anterolaterais com acesso intracavitário, com idade entre 18 e 75 anos. Os critérios de exclusão foram os seguintes: pacientes que apresentavam metástases ósseas, pacientes com quadro doloroso prévio em decorrência de outras comorbidades, e pacientes dependentes de drogas, analgésicos opioides ou qualquer outra substância que afetasse sua sensibilidade à dor.

Os pacientes foram randomizados e distribuídos em dois grupos conforme o procedimento cirúrgico realizado. A randomização foi realizada por meio de *web-based randomization*. O grupo SP foi composto por pacientes submetidos a fechamento torácico através de SP, e o grupo ST foi composto por pacientes submetidos a fechamento torácico por meio de ST.

No grupo SP**, **a aproximação costal foi realizada através de SP, que consiste na passagem do fio de sutura contornando a quinta costela rente a sua borda superior e contornando a sexta costela distante da sua borda inferior, aproximando-as e fechando a toracotomia (Figura 1).

**Figura 1:** Técnica de fechamento por sutura pericostal.

Nos pacientes do grupo ST, o fechamento foi realizado através de fios transcostais. Marcou-se no periósteo a posição da passagem dos fios de sutura com um bisturi elétrico. Em seguida, realizaram-se as perfurações do quinto e sexto arcos costais com uma broca de 7 mm de diâmetro, acionadas por rotação com um micromotor odontológico LB100 (Beltec, Araraquara, Brasil; Figuras 2A e 2B).

**Figura 2:** Em A, perfuração da costela através da broca. Em B, aproximação do quinto e sexto arco costal após a passagem do fio de sutura pelos forames realizados pela broca.

Foram realizadas quatro perfurações equidistantes em cada costela. Os fios de sutura foram passados pelos orifícios e procedeu-se ao fechamento transcostal (Figura 2B). Em todos os fechamentos, utilizou-se um fio de sutura sintético e absorvível de VICRYL**(r)** (poliglactina 910) com cobertura (Ethicon Endo-Surgery, Inc. Cincinnati, OH, EUA) no. 1 com uma agulha circular de 40 mm.

As variáveis avaliadas foram dor no período pós-operatório e função pulmonar por espirometria no 21º dia pós-operatório (DPO) e no 60º DPO para comparações com os valores obtidos no pré-operatório. Segundo o protocolo do estudo, a avaliação da dor foi realizada até o 10º DPO, assim como no período pós-operatório tardio (21º DPO e 60º DPO).

Para a avaliação da dor, foram utilizados uma escala visual analógica (EVA) unidimensional e o questionário de dor McGill.^(^ 13 ^)^ A EVA é uma escala graduada de 0 a 10, no qual o terapeuta questiona o seu paciente quanto a sua intensidade de dor, sendo que 0 significa ausência total de dor e 10 significa o nível de dor máxima sentida. O questionário de dor McGill avalia a dor em quatro dimensões distintas (sensorial, afetiva, avaliativa e mista), baseado em palavras, denominadas descritores, as quais os pacientes selecionam para descrever sua dor.^(^ 13 ^)^ O paciente é orientado a escolher, dentre 20 grupos de descritores, aqueles que melhor descrevem a sua dor no momento da avaliação.^(13) ^Os primeiros 10 descritores estão relacionados à dimensão sensorial da dor. Os descritores 11-15 relacionam-se à dimensão afetiva da dor. O descritor 16 refere-se à dor de maneira avaliativa, enquanto os descritores 17-20 representam uma classe mista de palavras alternativas.^(^ 13 ^)^

A espirometria foi realizada segundo os critérios da *American Thoracic Society* de 1995 e da Sociedade Brasileira de Pneumologia e Tisiologia.^(^ 14 ^)^ Em um ambiente estável, o paciente sentava-se em posição confortável e, usando um obturador nasal, realizava uma expiração máxima forçada a partir da CPT até o VR. Dessa forma, foram aferidos CVF, VEF_1_, relação VEF_1_/CVF e PFE.^(^ 14 ^)^

Os dados individuais foram expressos em média e seu erro padrão. A análise estatística foi realizada com o uso do programa GraphPad Instant (GraphPad Software, San Diego, CA, EUA). As variáveis categóricas (gênero, etnia, diagnósticos clínicos e procedimentos cirúrgicos) foram avaliadas pelo teste do qui-quadrado. Para as variáveis numéricas (espirometria), utilizou-se o teste t de Student na comparação dos resultados entre os grupos SP e ST e *one-way* ANOVA na comparação dos resultados antes e após a cirurgia dentro do mesmo grupo. Para a análise da dor quantificada pela EVA, utilizou-se o teste t de Student, enquanto, para a avaliação da dor pelo questionário de dor McGill, utilizou-se o teste de Mann-Whitney.^(^ 15 ^)^ Para a rejeição da hipótese de nulidade, adotou-se o nível de significância p < 0,05.^(^ 15 ^)^

## Resultados

Foram incluídos no estudo 31 pacientes, sendo 16 e 14 randomizados para os grupos SP e ST, respectivamente. Somente 1 paciente do grupo ST não retornou para a reavaliação espirométrica, sendo esse excluído da pesquisa. A Tabela 1 apresenta a caracterização da amostra.

**Tabela 1:** Caracterização da amostra e procedimentos realizados nos grupos estudados.a

Variáveis	Grupos		p
	Sutura pericostal	Sutura transcostal	
	(n = 16)	(n = 14)	
Idade, anos^b^	53,6 ± 3,4	48,9 ± 4,4	0,39
Gênero			
Masculino	11 (68,8)	7 (50,0)	0,50
Feminino	5 (31,3)	7 (50,0)	
Etnia			
Branca	14 (87,5)	10 (71,4)	0,14
Afrodescendente	2 (12,5)	3 (21,4)	
Amarela	0 (0,0)	1 (7,1)	
Cirurgias realizadas			
Lobectomia	9 (56,35)	8 (57,1)	0,12
Bilobectomia	4 (25,0)	3 (21,4)	
Segmentectomia	3 (18,8)	3 (21,4)	

a Valores expressos em n (%), exceto onde indicado

b Valores expressos em média ± ep.

O diagnóstico dos pacientes nos grupos SP e ST, foram, respectivamente, os seguintes: adenocarcinoma, em 10 e 8 pacientes; carcinoma epidermoide, em 3 e 3; carcinoma de pequenas células, em 2 e 3; e sequelas de tuberculose, em 1 e 0. A lobectomia foi o procedimento cirúrgico mais comumente realizado (Tabela 1). O tempo médio de duração do procedimento cirúrgico foi de 271,5 ± 25,7 min para o GP e de 250,3 ± 23,4 min para o grupo ST (p = 0,88).

Calculou-se a média numérica da intensidade da dor para cada DPO. Em ambos os grupos, observou-se o decréscimo da intensidade dolorosa durante a evolução. Observou-se uma maior intensidade dolorosa nos pacientes do grupo SP do que nos do ST desde o pós-operatório imediato, e essa diferença foi estatisticamente significante até o 7º DPO (p < 0,0001; Figura 3A). Nos pacientes do grupo ST, a dor era mínima ou inexistente em torno do 7º DPO, enquanto os pacientes do grupo SP ainda referiam dor moderada naquele momento. No grupo SP, a dor somente foi relatada como mínima no 60º DPO.

**Figura 3:** Comparação da média da avaliação da intensidade da dor em diversos dias pós-operatórios nos grupos pericostal (GP) e transcostal (GT). Em A, escala visual analógica. Em B, somatória dos números de descritores selecionados pelos pacientes no questionário de dor McGill. Em C, somatória dos escores pelo questionário de dor McGill.

Após a aplicação do questionário de dor McGill, para cada grupo de descritores avaliados (afetivo, sensitivo, avaliativo e misto), foram calculadas e comparadas as médias dos números dos descritores escolhidos e as médias da somatória dos índices desses descritores para cada dia de avaliação. Essa avaliação foi realizada do 1º ao 10º DPO e repetida no 21º DPO e no 60º DPO. Quando comparou-se o número total de descritores escolhidos pelos pacientes nas quatro categorias avaliadas, foi observado um número maior de descritores no grupo SP em relação ao número total no ST. A intensidade de dor pós-operatória avaliada por essa escala demonstrou que a dor foi maior nos pacientes do grupo SP. Essa diferença foi estatisticamente significante entre os dois grupos até o 10º DPO (p < 0,01; Figura 3B). Quando comparou-se a somatória dos valores dos descritores escolhidos pelos pacientes nas quatro categorias de descritores avaliados, foi observada que a somatória desses valores foi superior no grupo SP em relação ao ST, nos primeiros 10 DPO, sendo essa diferença estatisticamente significante (p < 0,001; Figura 3C).

A avaliação da função pulmonar por espirometria foi realizada em três momentos distintos: no período pré-operatório, no 21º DPO e no 60º DPO. O procedimento cirúrgico implicou em reduções de valores espirométricos em ambos os grupos, pois o procedimento cirúrgico causa uma ressecção parcial do pulmão; no entanto, segundo a análise estatística, houve uma homogeneização da amostra em relação ao tipo de cirurgia realizada (p = 0,12). A Tabela 2 demonstra os valores espirométricos em cada momento do estudo nos dois grupos. Os valores de CVF, VEF_1_ e PFE foram significantemente menores no período pós-operatório que no pré-operatório no grupo SP, enquanto não houve diferenças significantes desses valores no ST.

**Tabela 2:** Resultados espirométricos no pré-operatório e no 20º e 60º dia pós-operatório nos grupos estudados.a

Variáveis	Momentos	GSP	p	GST	p
CVF, L	Pré	3,00 ± 0,30	0,007	2,85 ± 0,20	0,14
	21º DPO	2,10 ± 0,10		2,38 ± 0,20	
	60º DPO	2,26 ± 0,10		2,61 ± 0,30	
					
VEF_1_, L	Pré	2,48 ± 0,10	0,01	2,33 ± 0,30	0,28
	21º DPO	1,72 ± 0,10		1,91 ± 0,30	
	60º DPO	1,89 ± 0,10		2,13 ± 0,30	
					
PFE, L/s	Pré	5,96 ± 0,50	0,02	5,30 ± 0,60	0,29
	21º DPO	4,03 ± 0,40		4,41 ± 0,50	
	60º DPO	4,80 ± 0,50		5,19 ± 0,70	
					
VEF_1_/CVF, %	Pré	83,4 ± 2,0	0,71	79,8 ± 4,0	0,51
	21º DPO	82,5 ± 2,0		83,2 ± 3,0	
	60º DPO	84,1 ± 2,0		81,7 ± 3,0	

GP : grupo sutura pericostal

GT : grupo sutura transcostal

Pré : pré-operatório

DPO : dia pós-operatório

a Valores expressos em média ± ep

## Discussão

Corroborando o nosso interesse pelo tema, no decorrer do presente estudo foi publicada uma revisão sistemática sobre as técnicas de sutura do espaço intercostal em toracotomias e suas relações com a dor pós-toracotomia.^(^ 16 ^)^ Os autores daquela revisão, utilizando a base de dados da Biblioteca Cochrane Plus através das palavras-chaves "dor", "toracotomia" e "sutura", levantaram 174 publicações que vinculavam a técnica cirúrgica empregada à dor pós-operatória. Desse total, 11 publicações preencheram os critérios de seleção estabelecidos e, desses, 6 comparavam a técnica de fechamento do espaço intercostal com a dor pós-toracotomia, sendo apenas 4 estudos aleatorizados. Concluíram os revisores que seria necessário maior evidência científica sobre determinados aspectos técnicos da sutura dos espaços intercostais e a dor pós-toracotomia. Assinalaram ainda que só se poderia estabelecer recomendações a esse respeito com o desenvolvimento de estudos prospectivos aleatorizados que permitissem comparar especificamente as distintas variantes das técnicas de fechamento de toracotomias descritas na literatura e sua relação com a dor pós-toracotomia. Entretanto, um aspecto que a revisão deixou claro para a redução da dor pós-toracotomia, e que deve ser comum a todas as técnicas de fechamento, é o enfoque na preservação dos nervos intercostais.

Entre as variáveis selecionadas no presente estudo a dor foi a que apresentou maior dificuldade do ponto de vista avaliativo, pois trata-se de uma variável contínua e difícil de ser quantificada. Além de ser um sintoma, é uma experiência subjetiva e acaba sofrendo a influência de diversos fatores, tais como fatores ambientais, emocionais, comportamentais e sociais. Por esse motivo, utilizaram-se duas ferramentas padronizadas (EVA e questionário de dor McGill).^(^ 13 ^)^

O estudo prospectivo de maior casuística pertinente a esse assunto incluiu 280 pacientes submetidos a toracotomias posterolaterais, distribuídos em dois grupos: suturas transcostais foram realizadas em 140 pacientes, e suturas pericostais foram realizadas em 140.^(^ 10 ^)^ A variável dor foi avaliada através de uma escala numérica de dor e questionário de dor McGill. Esses instrumentos foram aplicados na segunda semana pós-operatória, assim como no primeiro, segundo e terceiro mês pós-operatório. Os autores concluíram que, nos pacientes tratados com ST, a intensidade de dor foi menor do que nos pacientes submetidos à técnica de fechamento pericostal. Apesar de aquele estudo não ser aleatorizado, ele apresentou um nível de evidência consistente para recomendar o uso da ST no fechamento de toracotomias.^(^ 10 ^)^

Em um estudo experimental em cães, avaliou-se a dor no pós-operatório imediato após a toracotomia em 13 animais.^(^ 17 ^)^ Sete animais tiveram o fechamento realizado por SP rente à borda inferior da costela inferior, comprimindo o feixe neurovascular (caudal), e 6 cães foram submetidos a fechamento por ST**.** A dor foi avaliada através da escores de limiar da dor, baseados em parâmetros como FC e FR, por um período de 24 h. O estudo demonstrou que a dor foi significativamente inferior no grupo tratado com ST.^(^ 17 ^)^ Embora tal estudo experimental adotara uma metodologia semelhante em relação à técnica cirúrgica empregada, mostrando-se de muito valor para evitar a compressão e a lesão do nervo intercostal, teve como limitação a avaliação da dor apenas no pós-operatório imediato.^(^ 17 ^)^

No presente estudo, foi possível verificar através da EVA e do questionário de dor McGill que os pacientes do grupo ST apresentavam menor intensidade de dor em relação aos pacientes do grupo SP; esses resultados são semelhantes aos resultados apresentados em estudos anteriores.^(^ 10 ^,^ 17 ^)^

Estudos anteriores^(^ 12 ^,^ 18 ^,^ 19 ^)^ também utilizaram a ST no fechamento de toracotomias; porém, houve variações na técnica de abertura do espaço intercostal durante a abordagem da cavidade pleural, de modo que seus resultados não são comparáveis aos resultados do presente estudo e aos de um outro estudo,^(^ 10 ^)^ no qual foram utilizadas variações técnicas na execução da toracotomia através da liberação de retalhos musculares intercostais para a proteção do feixe neurovascular do afastador torácico. Desse modo, a avaliação do limiar da dor no pós-operatório ficou prejudicada na comparação dos grupos de fechamento transcostal e pericostal porque houve intervenções diferentes.

Sabe-se que a utilização de afastadores de Finochietto durante a abertura torácica é responsável por grande parte da dor após o procedimento cirúrgico. Em nosso estudo, levamos esse fato em consideração, motivo pelo qual foi utilizada a mesma metodologia de abertura em ambos os grupos, ou seja, sem variações na técnica da abertura, como sugerida anteriormente por outros autores.^(^ 12 ^,^ 16 ^)^

Ainda com relação à dor, foi observado no presente estudo que os pacientes do grupo SP utilizaram um elevado número de descritores para caracterizar sua dor no período pós-operatório - em média 11 descritores no 1º DPO, com um escore médio de 26. Esse fato também foi observado em um estudo prospectivo^(6) ^que comparou a dor, através do questionário de dor McGill, de 40 pacientes submetidos a toracotomia posterolateral e a esternotomia. O número médio de descritores apresentados nos pacientes do grupo que realizou toracotomia posterolateral foi de 16, com escore médio de 30, valores esses muito próximos aos encontrados no presente estudo.

Com relação à função pulmonar, observou-se que os pacientes submetidos ao fechamento torácico convencional (grupo SP) apresentavam valores de CVF, VEF_1_ e PFE significantemente menores no 21º DPO quando comparados com seus resultados no pré-operatório. Esses resultados são historicamente esperados no pós-operatório de toracotomias e foram semelhantes aos relatados em estudos anteriores.^(^ 20 ^,^ 21 ^)^

Em um estudo anterior,^(^ 19 ^)^ foi investigada a função pulmonar em 16 pacientes após a realização de toracotomias amplas. As espirometrias foram realizadas no 14º DPO. Os autores observaram que CVF, VEF_1_ e PFE eram significativamente menores em relação aos valores pré-operatórios. ^(^ 19 ^)^ A recuperação dos pacientes em relação a essas variáveis se deu pela melhora na capacidade ventilatória, pela redução da lesão cirúrgica da parede torácica e pelo alívio da dor.

Em um estudo prospectivo com 33 pacientes submetidos à cirurgia torácica, foi avaliada a repercussão da ressecção pulmonar sobre a função pulmonar de pacientes com câncer de pulmão submetidos a toracotomias.^(^ 21 ^)^ As espirometrias foram realizadas no pré-operatório e no 6º mês pós-operatório. Foram verificadas reduções estatisticamente significantes nos valores de VEF_1_, PFE e CVF quando comparados com os valores no pré-operatório. Tais resultados eram esperados e estão relacionados ao impacto direto da ressecção cirúrgica e à dor no período pós-operatório.^(^ 21 ^)^

No presente estudo, por se tratar de procedimentos cirúrgicos que implicam em ressecção de parênquima pulmonar, espera-se uma redução nas variáveis espirométricas; no entanto, os procedimentos executados em ambos os grupos foram bastante similares e houve concordância entre uma menor intensidade de dor pós-operatória no grupo ST com uma menor queda nos valores da CVF, VEF_1_ e PFE. No grupo SP, cujo registro de dor foi mais intenso, as reduções dos valores espirométricos foram maiores.

Em conclusão, os pacientes submetidos ao fechamento de toracotomias posterolaterais e anterolaterais por ST apresentaram significante redução da intensidade de dor pós-operatória, tanto imediata como tardia, quando comparados aos pacientes submetidos à técnica pericostal. Além disso, os pacientes do grupo ST apresentaram menores reduções nos parâmetros espirométricos. Dessa forma, a técnica de fechamento do espaço intercostal por ST é recomendada por apresentar vantagens sobre a técnica pericostal.
